# Targeting CD44 and other pleiotropic co-receptors as a means for broad inhibition of tumor growth and metastasis

**DOI:** 10.1007/s10585-024-10292-4

**Published:** 2024-05-18

**Authors:** Lisa-Marie Mehner, Leonel Munoz-Sagredo, Steffen Joachim Sonnentag, Sven Máté Treffert, Véronique Orian-Rousseau

**Affiliations:** 1 Institute of Biological and Chemical Systems – Functional Molecular Systems, Karlsruhe Institute of Technology, Karlsruhe, Germany; 2 School of Medicine, Universidad de Valparaiso, Valparaiso, Chile

**Keywords:** Cancer, Metastasis, CD44, Co-receptors, Pleiotropicity

## Abstract

Although progress has been made in the treatment of cancer, particularly for the four major types of cancers affecting the lungs, colon, breast and prostate, resistance to cancer treatment often emerges upon inhibition of major signaling pathways, which leads to the activation of additional pathways as a last-resort survival mechanism by the cancer cells. This signaling plasticity provides cancer cells with a level of operational freedom, reducing treatment efficacy. Plasticity is a characteristic of cancer cells that are not only able to switch signaling pathways but also from one cellular state (differentiated cells to stem cells or vice versa) to another. It seems implausible that the inhibition of one or a few signaling pathways of heterogeneous and plastic tumors can sustain a durable effect. We propose that inhibiting molecules with pleiotropic functions such as cell surface co-receptors can be a key to preventing therapy escape instead of targeting bona fide receptors. Therefore, we ask the question whether co-receptors often considered as “accessory molecules” are an overlooked key to control cancer cell behavior.

Although the role of growth factors was characterized by developmental biologists more than half a century ago, it was much later that their receptors were used as targets for anti-cancer therapy. With the arrival of the current millennium, the first monoclonal antibodies (trastuzumab, cetuximab) and small molecule inhibitors (gefitinib, imatinib) to block some of these receptors reached clinical application. Simultaneously, the completion of the Human Genome Project facilitated the recognition of diverse somatic mutations in cancer and boosted the development of molecular targeted therapies. By 2020, regulatory agencies had approved 89 small molecule inhibitors [1] and 23 monoclonal antibodies [2] against ligands, receptors and membrane-bound proteins. Monoclonal antibodies are highly target-specific but act exclusively on extracellular targets, while small-molecule inhibitors can be less selective but can target intracellular targets such as kinases involved in signal transduction [3].

These developments led to a conceptual framework based on blocking aberrantly activated receptors or downstream kinases in cells that generate signals that influence cancer hallmarks of sustained proliferation signaling, resistance to cell death, invasiveness and immune evasion [4]. The use of genomic or mRNA sequencing and high-throughput epigenetic profiling has revealed modifications that can be specifically targeted, thus encouraging the idea of personalized medicine. The cancer research and clinical oncology communities, funding agencies, and industry have embraced this strategy, which accelerated the discovery of multiple actionable mutations. The development of agents to target them has significantly impacted the survival of specific groups of cancer patients. However, this strategy still faces important challenges [5, 6].

One relevant challenge is the functional redundancy of signaling pathways. It is a manifestation of alternative elements that contribute to a gene regulatory network in which the loss-of-function of one element can be compensated for or substituted by another. It is likely a product of evolution that confers robustness to cell communication systems of multicellular organisms [7–9]. Once ligands bind to their receptors, a chain of reactions clearly stands out from the background noise of spontaneous biochemical reactions. However, they are interconnected in the form of networks, generating several outputs [10]. Furthermore, cells conjugate several extracellular cues simultaneously, which are then integrated through these networks. The connection nodes of these networks – i.e. signaling relays that integrate two or more signaling pathways to merge towards downstream effectors – are responsible for what has been called “adaptive resistance” to molecular targeted therapies [3, 5, 6]. This may lead to resistance after an initially positive response. For example, in the treatment of non-small cell lung cancer (NSCLC) and colorectal cancers with anti-EGFR agents (erlotinib, gefitinib), resistant tumors exhibit a significantly higher proportion of MET receptor amplifications, another receptor tyrosine kinase (RTK) for the hepatocyte growth factor (HGF), that is frequently present in those tumor microenvironments [11–13].

The phenomenon of adaptive resistance can be approached with the use of sequential targeted therapies, choosing a new agent upon resistance development and disease progression, or by simultaneously targeting multiple pathways, also called horizontal pathway inhibition [14, 15]. Given the molecular diversity of cancers, the intra-tumoral heterogeneity and the multiplicity of resistance mechanisms, the best approach is still an open question and will unlikely correspond to a single general strategy. A fore mentioned, approved single-targeted agents have been accumulating in the last decades and novel agents demonstrating specific advantages are likely to broaden the spectrum of choices for sequential treatments. Due to the evolution of tumors that develop resistance, the rational choice of the next agent may require new biopsies, that is not always possible. Sampling is also not a trivial issue in heterogeneous tumors. Though still in its infancy, liquid biopsies may prove to be highly useful for this approach [16, 17].

In parallel, combinations of these agents for specific cancers have been approved and are part of a growing list of clinical trials. A central issue for this approach is the increase in toxicity upon combination. When a combination of two agents is used to target different pathways, in approximately half of the clinical trials, doses had to be lowered due to toxicity, thus decreasing the efficacy of inhibition. This was especially clear for combinations that included the inhibition of mammalian target of rapamycin (mTOR) [18]. An observational study involving 160 patients with advanced metastasis recommends not to use trametinib (a MEK inhibitor) and everolimus (a mTOR inhibitor) concomitantly, even at lower doses [19]. Rational approaches to find the best combinations, is a field under development [20].

Similar to specific agent combination therapies, less specific signaling pathway inhibition has also been developed and approved for some cancers resistant to conventional therapies. Since the first multi-kinase inhibitor (MKI), sorafenib, was approved for the treatment of hepatocellular, renal cell and thyroid carcinomas [15], twelve agents are currently available. For example, sunitinib inhibits multiple RTKs including VEGFR-1, VEGFR-2, c-KIT, and PDGFR-α, and sorafenib that apart from the effects on RTKs, blocks intracellular kinases including wild-type and mutant BRAF. They have a predominant antiangiogenic effect, but also affect cell proliferation and cell survival in in vitro systems [21]. However, they face similar toxicity limitations as combination therapies, with adverse effects reported in virtually all patients under MKI regimes [3, 22]. This underscores the fact that inhibition targeted at specific multiple components of hyperactive pathways in cancer cells also affects the normal-functioning pathways in non-cancerous cells. As a result, the use of this strategy may be subject to limits similar to those imposed on non-specific cytotoxic chemotherapeutic agents.

Reflections on the need of multiple pathway inhibition for the advancement of precision medicine and the limitations imposed by toxicity on non-cancerous cells, prompted us to explore in this review the possible role of the inhibition of CD44, a pleiotropic co-receptor of multiple cell surface receptors. These include RTKs, serine-threonine kinase receptors (e.g. TGF-β receptors), G protein-coupled receptors (GPCRs, like chemokine receptors), and the Wnt signalosome, that may be highly active in cancer cells [23]. Blocking of CD44 in cells in vitro as well as in in vivo experiments, has shown a profound inhibition of signal transduction of its associated receptor [24–28]. We reckon that this evidence places CD44 inhibition in a similar position to current multitargeting strategies with regards to toxicity. However, we propose that the rich alternative splicing of this co-receptor and splicing switches triggered by pathological conditions like cancer and inflammation, together with the possibility of specific isoform inhibition, may act as regulatory hubs both in cancer cells and in tumor stromal cells, avoiding non-tumor cells. Therefore, we hypothesize, that this phenomenon may confer cell-type specificity to this approach. Interestingly, CD44 and co-receptors in general have been rarely described as being mutated or aberrantly expressed in cancer, adding the advantage of target stability. Given the pleiotropicity of co-receptor molecules in general (Fig. 1), we further explored the availability of evidence on the impact of the inhibition of co-receptors other than CD44. Our search retrieved encouraging experimental evidence about co-receptors as targets, though rather scarce and still restricted to in vitro systems and in vivo models, with few cases that have made it into clinical trials to date.

**Fig. 1:**
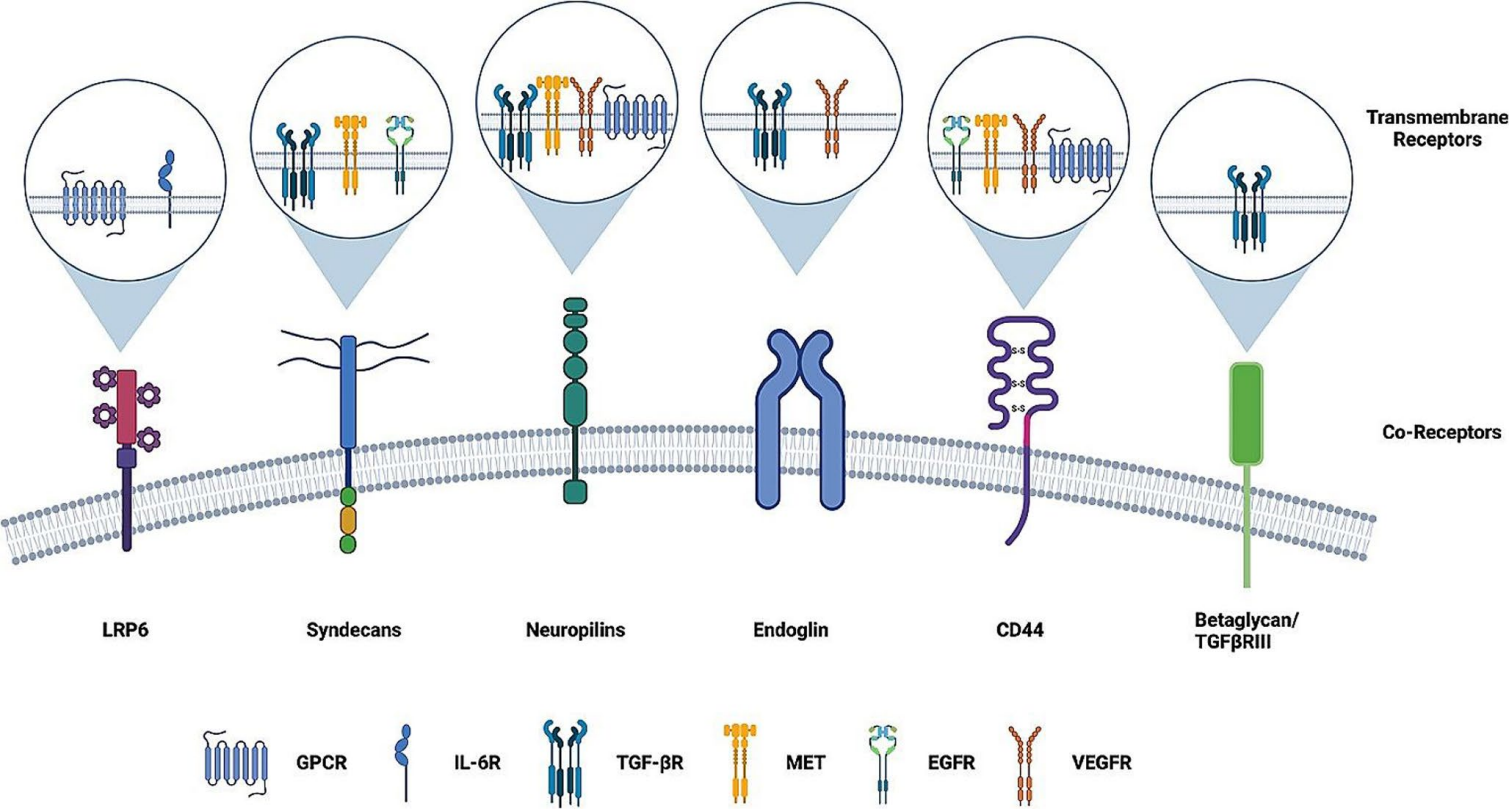
Schematic illustration of co-receptors with their corresponding partner receptors. Figure created with BioRender.com

## Co-receptors and their involvement in signaling

The term co-receptor describes a limited number of proteins that collaborate with cell surface receptors to promote their activation through different mechanisms, thereby impacting on their signal transduction from extracellular cues into the cell [29–32]. They can be indispensable for activating the receptors or augmenting their signal transduction efficiency through mechanisms acting in the receptor’s extracellular or the cytoplasmic domains. At these levels, they may interact with ligands and intracellular signaling or adaptor molecules. They are involved in the control of cell behavior in the physiological context as well as during disease. These pleiotropic functions can be used advantageously to influence cancer cell behavior. From this perspective, molecules like CD44, ICAMs, syndecans, LRP5-6, or TGF-β co-receptors such as endoglin, β-glycan and neuropilin are interesting target molecules. For example, CD44 controls major signaling pathways such as those downstream of RTKs, receptor protein serine-threonine kinases, Wnt/β-catenin and GPCRs signaling [33]. Interestingly, when knocked out in animal models, the physiological effects observed in the case of CD44 are mild [25, 34], but its impact in the pathological context is strong [33]. As targets for cancer treatment, co-receptors might provide potential therapeutic specificity.

Here we review published evidence on the potential utility of these protein families and in particular of CD44 as potential cancer therapeutic targets.

### a. CD44, a signaling platform impacting metastasis

**Fig. 2:**
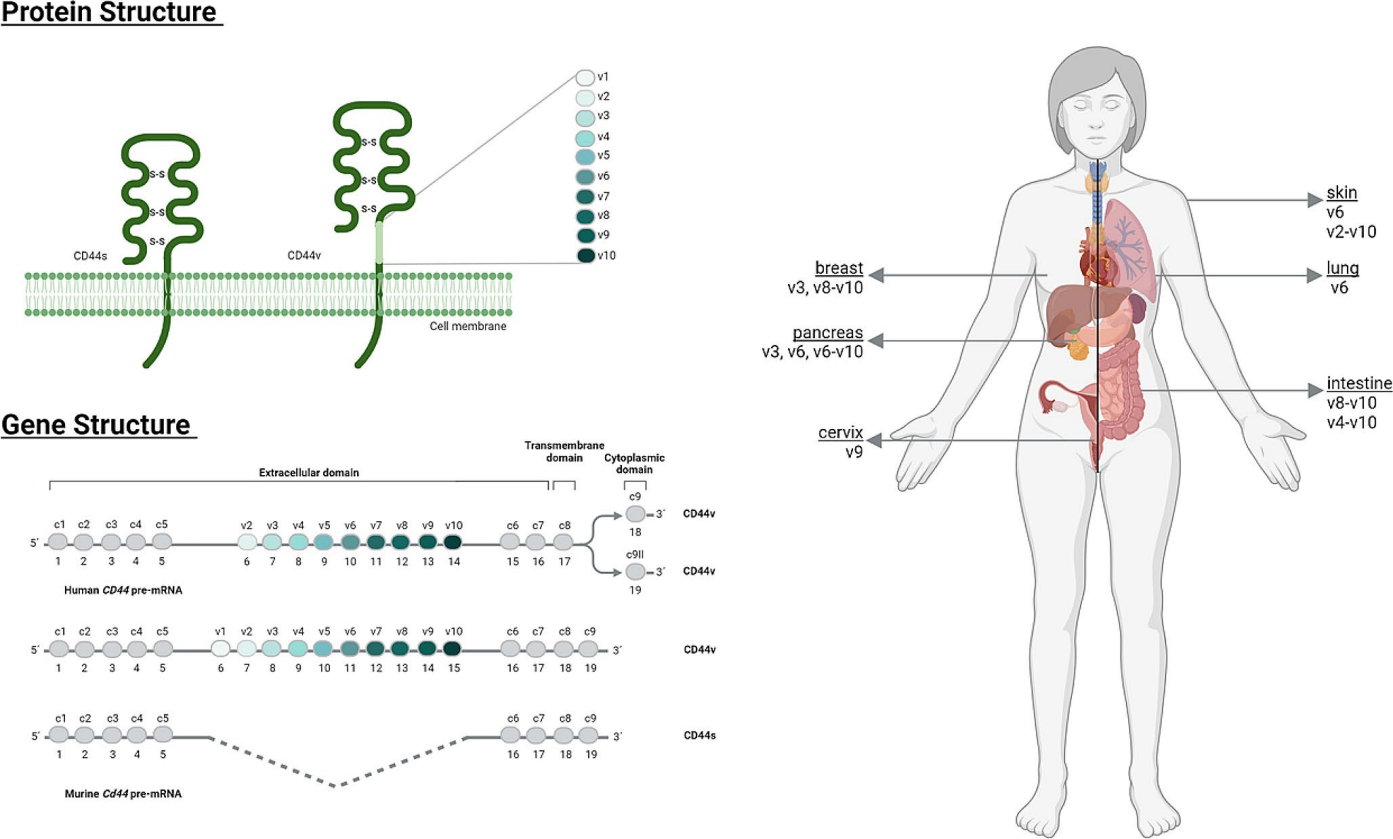
Schematic representation of the CD44 family of proteins, the gene structure and the expression pattern of specific isoforms. Figure created with BioRender.com

The term CD44 designates a family of isoforms of transmembrane glycoproteins that take part in major cellular processes including migration, differentiation, proliferation, and survival [35] (Fig. 2). The human *CD44* gene encodes 19 exons, ten of which are expressed in all CD44 isoforms, whereas the other nine exons (known as variant exons) can be included or excluded theoretically in all possible combinations by alternative splicing (Fig. 2). The heterogeneity of these proteins is further enhanced by extensive glycosylation. CD44 standard (CD44s), the smallest and most ubiquitously expressed isoform of the family, does not include any variant exon products. Other isoforms, such as the CD44v6 isoforms containing either the exon v6 product alone or in combination with other variant exon products, show a restricted pattern of expression (Fig. 2). The CD44v6-containing isoforms are expressed in proliferative tissues such as the skin or the intestinal epithelium. CD44v8-v10 is known as the epithelial isoform. CD44v2-v10, a long isoform containing all variant exon products, is highly expressed in human skin. Of note, the v1 exon is not expressed in human cells.

CD44 isoforms, such as CD44v6, have been shown to act as co-receptors for the RTKs MET, VEGFR-2 and EGFR. Their respective ligands, HGF, VEGF, EGF or neuregulin, recruit CD44v6 [36]. CD44v6 appears to provide the same functions to the various growth factors and growth factor receptors. Indeed, the ectodomain of CD44v6 directly binds to HGF and VEGF and is required for activating the corresponding receptors. The cytoplasmic domain of CD44v6 recruits ezrin-radixin-moesin (ERM proteins) in order to bind to the actin cytoskeleton, presumably providing a signaling platform necessary for signal transduction. Although direct binding of EGFR family ligands to CD44v6 has not yet been shown, the same dual mechanism appears to take place. Indeed, the interplay between CD44v6 and members of the EGFR family has been shown to be involved in breast cancer progression [37]. The HGF/MET/CD44v6 signaling supports pancreatic cancer tumor growth and metastasis [25]. In various mouse models of pancreatic cancer, including the LSL-Kras^G12D/+^;LSL-Trp53^R172H/+^;Pdx-1-Cre model, inhibition of CD44v6 by means of a CD44v6 peptide blocked tumor progression and metastasis. Most importantly, a comparison between the CD44v6 peptide activity and crizotinib, a MET/ALK inhibitor, showed a stronger effect of the CD44v6 peptide, thus indicating more potential for inhibitors with a broader spectrum of action [25]. In addition, previously established spontaneous metastases underwent apoptosis in animals orthotopically injected with rat or human pancreatic cancer cells and treated with the v6 peptides after the appearance of metastases. Two additional peptides, identified through a phage-display peptide library screen, inhibited MET activation when directed against CD44v6. These peptides also inhibited metastasis of human and mouse breast cancer cells in experimental and spontaneous metastasis models, respectively [38].

The heparan sulfate modifications exposed on the extracellular surface of CD44v3 isoforms are thought to mediate the activation of FGFR by its ligand FGF. This interplay between CD44v3/FGF/FGFR was shown to be implicated in the proliferation of mesenchymal cells underlying the apical ectodermal ridge during limb development [39]. FGF, together with HB-EGF, is a heparin-binding growth factor. In the presence of HB-EGF, a study in breast cancer cells showed that CD44v3 is required for activation of EGFR [37]. CD44v3 also binds to TrkA upon induction with NGF [40]. Peptides from the exon v3 sequence impaired NGF-RhoA activation and dependent clonogenicity and migration/invasion. The relevance of this interaction was demonstrated in a breast cancer model, where the v3 peptides blocked tumor growth and metastasis.

Other CD44 isoforms such as CD44s have been shown to facilitate dimerization of ErbB receptors [41]. More recently, our group has identified a complex between CD44s and TGFβRs, which is important in the activation of stellate cells in the pancreatic tumor microenvironment (our own unpublished data).

Besides RTKs, other types of cell surface receptors recruit CD44 isoforms in order to function. This is the case of CXCR4, a GPCR that binds to the chemokine CXCL12. Upon induction with low molecular weight HA, a complex between CD44 and CXCR4 is formed that induces angiogenesis [42]. CD44 is in direct contact with CXCR4, as demonstrated by split-Venus bimolecular fluorescence complementation performed under induction with CXCL12 [28].

The Wnt pathway also recruits CD44 isoforms. CD44 is directly involved in the function of the Wnt signalosome by enhancing its signal transduction [26]. It is in direct contact with LRP6 as shown by FLIM-FRET [27]. The membrane localization of LRP6 depends on CD44, which also influences the stability of the LRP6 mature form. Upon induction with Wnt, CD44 can be found in close vicinity to Axin-2 and Dishevelled. Since *CD44* is also a Wnt target gene, its role in the Wnt signalosome means that CD44 exerts a positive feedback loop that can be beneficial in the case of intestinal regeneration. In colorectal cancer, however, the CD44/Wnt interplay might support tumorigenesis, and is therefore a potential target for Wnt signaling modulation.

The importance of *CD44* splicing in tumor progression was clearly demonstrated in a study from the group of Riccardo Fodde, which showed that epithelial-mesenchymal transition (EMT) and the reverse process of mesenchymal-epithelial transition might be regulated by a splicing switch between CD44v6 to CD44s and other proteins, including NUMB2/4. These spliced isoforms were subsequently shown to promote invasion and metastasis [43]. The latter study also showed the involvement of the splicing factor ESRP1 and other RNA-binding proteins downstream of Zeb1, a master regulator of EMT, to induce these splicing switches.

Resistance to chemotherapy has been associated with various CD44 isoforms. However, it is still not entirely clear whether a specific CD44 signature is related to this process. For example, in breast cancer, aberrant *CD44* splicing leading to a CD44^high^ state was shown to mediate resistance to PI3K inhibitors. In that case, HA was shown to induce CD44-dependent Src/ERK signaling that bypassed PI3K signaling by maintaining AKT and mTOR activation. In addition, increased estrogen receptor (ER)- dependent transcription observed in cells treated with a PI3K inhibitor was reduced upon downregulation of CD44. These data indicate that the crosstalk between CD44 and ER is instrumental for this resistance mechanism [44]. In the latter study, the overall CD44 expression was increased. In colorectal cancer, the resistance of cancer initiating cells (CICs) to FOLFOX therapy was shown to be dependent on the specific expression of CD44v6. Factors such as periostin and IL-17A secreted by cancer associated fibroblasts (CAFs) increased CD44v6 expression through Wnt3A production. The CD44v6-dependent crosstalk between CICs and CAFs sustained resistance to chemotherapy [45]. In another study, the same group demonstrated sustained activation of Wnt signaling by FOLFOX that induced splicing of CD44, thereby increasing CD44v6 expression. The findings suggested that the Wnt3a-CD44v6 axis promotes resistance in the presence of FOLFOX. The formation of CD44-LRP6 signalosomes in caveolin domains led to augmented FOLFOX efflux [46]. Furthermore, in pancreatic cancer, resistance to gemcitabine was associated with a specific CD44 expression pattern where CD44s is the dominant isoform. In that study, the insulin growth factor receptor (IGFR) was shown to induce a splicing switch in response to gemcitabine. Silencing of CD44 in gemcitabine-resistant cells suppressed EMT and restored E-cadherin expression [47], suggesting that inhibition of CD44 splicing might be interesting to investigate in the context of combination therapy.

In acute myeloid leukemia (AML), our results point to a decisive role for CD44 in induction of stemness and resistance to chemotherapy [28]. We have identified a function of CD44 in concert with CXCL12/CXCR4 in the resistance of AML cells to venetoclax, which, in combination with hypomethylating agents or low-dose cytarabine, may offer hope to patients deemed unfit for classical chemotherapy against AML. 20% of patients with AML remain refractory to this combination and develop resistance. We found that the interplay between CD44 and CXCR4 increases the stemness properties of the leukemic cells as well as their Mcl-1 production in order to evade the venetoclax treatment. In summary, CD44 appears to be an attractive molecular target for the modulation of several signaling pathways that drive important biological processes in cancer. Due to its rich and tightly regulated alternative splicing, pathology-associated isoforms of CD44 may provide specific targets that avoid negative consequences for normal cell physiology.

### b. Other examples of co-receptors

#### i) Syndecans

Syndecans are heparan-sulfated proteoglycans that interact with several other cell surface receptors and build signaling platforms comprising integrins and RTKs [48]. This family of proteins consists of four members: syndecan 1–4. The heparan sulfate moiety recruits multiple ligands such as Wnts, extracellular components such as fibronectin or growth factors such as FGF, which enables syndecans to participate in several signaling pathways. A study from the group of A. Rapraeger illustrated the function of such a supra-molecular complex containing syndecan-2 and -4, integrins α3β1 and α6β4, RON and ABL1 in EGFR-independent proliferation of triple negative breast carcinoma cells and head and neck cancer cells [49]. In normal epithelial cells, EGFR is associated with syndecan-4 and the integrins α3β1 and α6β4. In the two types of cancer cells mentioned above, the recruitment of syndecan-4 and RON and ABL1 bypasses EGFR and allows them to evade the EGFR-targeted therapy. Although the functions of these complexes still need to be addressed in vivo, these data support the idea that proteoglycans like syndecans organize protein networks involved in resistance to anti-RTK therapies. Supporting this notion, a peptide targeting the EGFR docking site for syndecan-4 was shown to induce cell cycle arrest. Most interestingly, in that case, cell cycle progression is dependent on the RON kinase and no longer depends on EGFR. However, the docking of EGFR to syndecan still plays a role. Another study by the same group unraveled the importance of the shedding of syndecan-1, which leads to formation of a ternary complex between VEGFR-2, VLA-4 (integrin α4β1) and sSdc1. The PKA-mediated phosphorylation of VLA-4 depends on another actor, CXCR4. This complex controls cell migration [50].

Another study showed that downregulation of syndecans, specifically syndecan-1, increases stemness and invasiveness. Indeed, decreased syndecan-1 expression induces integrin β1, FAK and Wnt signaling activation [51]. However, decreased levels of *Sdc1* expression impacted the formation of spheres in serum-free suspension cultures and induced larger tumors in vivo.

The contribution of syndecans to tumor progression is not limited to their expression on cancer cells. Syndecan-2, which is expressed on tumor-associated stromal cells in breast tumors, contributes to metastasis and immune evasion. This effect is achieved through the regulation of TGF-β signaling [52]. Interestingly, specific targets of TGF-β, such as CXCR4 and PDL-1, are controlled by syndecan-2. Indeed, a syndecan-2 peptide blocking syndecan-2 also decreased expression of CXCR4 and PDL-1, thereby diminishing the immunosuppressive properties of tumor-associated stromal cells.

These examples suggest that syndecans are active at an intersection between several pathways. This pleiotropic action could be advantageous for anti-cancer treatment, but the combination of associated receptors could also support tumorigenesis. The inhibition of syndecans thus requires careful analysis in various contexts.

#### ii) LRP5/6

The Wnt signaling pathway is involved in essential steps during development and many important biological processes, including tissue homeostasis and regeneration [53]. Therefore, it is not surprising that dysregulation of this signaling pathway has serious pathological consequences. For example, 80% of colorectal cancer (CRC) patients show a mutation in the *APC* gene, a key protein that has a fundamental role in regulating the Wnt pathway [54]. Thus, its mutation results in constant activation of the pathway. However, the impact of dysregulation of the Wnt pathway is not limited to downstream mutations. Several variants of the membrane protein LRP6, an essential co-receptor of the Wnt pathway, have also been linked to cancer development [55]. Interestingly, overexpression of LRP6 was observed in CRC, probably due to the hypermethylation of the LRP6 repressor *Necdin* but not the result of a mutation [56]. Yao et al. showed that the phosphorylation of LRP6 led to the rearrangement of the actin cytoskeleton via the RhoGTPases RhoA and Rac [57]. Cytoskeletal remodeling takes place during epithelial-mesenchymal transition (EMT) and is also dependent on nuclear β-catenin. The overexpression of LRP6 in the colon cancer cell lines LoVo and HCT116 showed a tendency towards an increased ability to migrate, indicating an increased invasive potential, which is crucial for both metastatic progression and development [57]. Furthermore, in the breast cancer cell line MCF-7, an shRNA-based knockdown of LRP6 resulted in reduced tumor growth [58].

In the absence of Wnt3a, LRP6, through its extracellular domain, can also influence the non-canonical signaling pathway mediated by FZD8 [59]. In an orthotopic breast cancer mouse model in which metastatic 4T1 cells and non-metastatic 168FARN cells were injected, a treatment using the LRP6 ectodomain (LRP6N) resulted in reduction of metastasis, suggesting that LRP6 might be a suitable target for treating metastatic cancer [59].

Whether inhibition of LRP6 can affect APC-inactivated colorectal cancer cells is still being debated, although it is agreed that its activation can enhance downstream signaling. Chen and He demonstrated that a CRISPR/Cas-based knockout of LRP6 does not lead to a reduction in the Wnt activity of APC-inactivated cells, suggesting that the Wnt signaling pathway is totally independent from its co-receptor [60]. However, Cabel and colleagues reported reduced total β-catenin and nuclear β-catenin after LRP6 knockdown by siRNA [61]. They explained the different results in comparison to Chen and He by the possible occurrence of compensatory effects following a knockout of LRP6.

The importance of LRP6 in tumorigenesis was also demonstrated by Ji and colleagues, who focused on Metastasis Associated Lung Adenocarcinoma Transcript 1 (MALAT1), the long non-coding RNA (lncRNA). MALAT1, like LRP6, is correlated with prognosis, survival and the metastatic process of CRC [62, 63]. After subcutaneous injection into nude mice, the shRNA-mediated downregulation of MALAT1 in LoVo CRC cells resulted in reduced tumor growth [63]. On the molecular level, the knockout of MALAT1 in LoVo CRC cells led to increased levels of miR-15 family members (miR-15s). Conversely, MALAT1 overexpression was accompanied by reduced miR-15s levels. Li and colleagues also showed that MALAT1 and miR15-s were interacting, thus functioning as competing endogenous RNA (ceRNA). Interestingly, one downstream target of miR-15s is LRP6. Analysis of miR-195 (miR-15s member) revealed a negative effect on the expression of LRP6, which showed increased expression at the metastatic sites and in recurrent primary tumors [64].

Nie and colleagues showed that the activation of LRP5 in turn drives cells towards a CSC phenotype and consequently supports chemoresistance of CRC cells through activation of both the Wnt/β-catenin and IL-6/STAT3 signaling pathways [65]. In their study, Nie and colleagues reported increased expression of LRP5 in CRC cell lines and CRC tissues, especially in advanced stages of CRC (stage III and IV). They also found increased expression of LRP5 in metastatic CRC tissues. The transcriptional activation of LRP5 in HCT116 CRC cells led not only to enhanced migratory capacity but also resulted in the formation of more and bigger tumor spheres. These results were also validated in vivo, whereby LRP5-overexpressing HCT116 cells formed bigger tumors after subcutaneous injection, suggesting the importance of LRP5 in tumorigenesis. In addition, the upregulation of LRP5 induced the expression of stemness markers like OCT3/4, ALDH1A1 and CD133. Coherently, CD133^+^ HCT116 cells had enhanced *LRP5* mRNA levels. Interestingly, HCT116 CD133^+^ and HCT116 cells, in which LRP5 was upregulated, showed enhanced mRNA levels of *IL6* and *STAT3*. In parallel, the Wnt pathway appeared to be more active in this cell population. Most importantly, CRC cells with activation of LRP5 were more resistant to chemotherapeutic agents such as platinum-based drugs. Silencing of LRP5 suppresses tumorigenicity of CRCs cells. Both the Wnt/β-catenin and the IL-6/STAT pathway appeared to be repressed upon silencing of LRP5. In parallel to the knockout of LRP5, proapoptotic genes were increased following cisplatin treatment, which suggests that LRP5 knockout sensitizes cells to cisplatin [65].

The studies cited above have shown that LRP5/6 – besides its well-known influence on the activity of the Wnt signaling pathway – also affects other biological processes through the inflammatory IL-6/STAT3 axis. This indicates that its targeting may have a broad effect. Indeed, several small molecules have been used to target this co-receptor (expression or phosphorylation) and have shown interesting effects on various types of cancer cells, including colorectal, pancreatic, gastric, breast, ovarian, and prostate cancer, as well as chronic lymphocytic leukemia [55]. However, their translation into clinical applications still needs to overcome the potential toxicity due to the impact on physiological Wnt functions.

#### iii) Neuropilin

The neuropilin family of transmembrane multifunctional non-tyrosine kinase receptors consists of two types, NRP1 and NRP2, both lacking a cytosolic kinase domain. Therefore, neuropilin primarily acts as co-receptor for various cell surface receptors or their respective ligands. They form holoreceptors, mostly in form of ternary complexes with receptor and ligand, with receptor tyrosine kinases (e.g. VEGFR-1/2 and MET), with serine threonine kinase receptors (e.g. the TGF-β receptor family) or with cell surface molecules (e.g. integrins or semaphorins) [66]. Aberrant expression of these co-receptors as well as their receptor partners can have pathological consequences. NRP1 and -2 have been implicated in the occurrence of metastases in various cancer types and both are generally involved in the proliferation, progression, invasion and migration of tumor cells as underlined by the following studies.

In prostate cancer, Vanveldhuizen reported that the mRNA level of* NRP1* was elevated ten-fold in the malignant phenotype [67]. Such overexpression has also been shown in cervical cancer cells. In oral squamous cell carcinoma and breast cancer, NRP2 was shown to positively influence the Wnt/β-catenin pathway [68]. In another study, NRP1 downregulation in pancreatic cancer cells led to decreased invasive properties and migratory potential [69]. EMT induction in triple-negative breast cancer was also impaired following downregulation of NRP1 expression. This was attributed to its function as a co-receptor for the TGF-β/Smad pathway [70]. Expression of NRP1 and NRP2 thus appears to be linked to various processes that are essential for metastasis formation.

Overall, neuropilins have been shown to influence resident stromal cells as well as immune cells recruited to the tumor microenvironment, supporting the notion that they have an impact on tumor progression and immunosuppression. Macrophages expressing NRP1 and NRP2 adopted an M2 phenotype [71, 72]. Downregulation of NRP expression in myeloid cells has been shown to decrease the availability of the mRNA of the immunosuppressive interleukin IL-10 [73]. Furthermore, NRP-expressing Tregs can interact through their NRPs with other immune cells such as dendritic cells, thus eliciting immunosuppressive reactions from these cells [74]. Additionally, the NRP1-semaphorin-4 A interaction maintains Tregs with high NRP1 expression in the microenvironment, that in turn further suppress effector T cells [75]. In mammary tumors, when recruited to hypoxic regions, tumor-associated macrophages with a high expression of NRP2, showed increased levels of MMP9, Tie-2, VEGF-A and HIF-1α [73]. This suggests a role in angiogenesis, another essential process in tumor progression and metastasis.

#### iv) TGF-β co-receptors (endoglin, β-glycan)

##### Endoglin

Endoglin, also known as CD105, interacts with TGFβRI and TGFβRII and plays a role in metastasis formation in various types of cancer mainly through its involvement in primary tumor angiogenesis. This 180 kDa transmembrane glycoprotein, primarily expressed on endothelial cells, exists in short (S-ENG) and long (L-ENG) protein isoforms that can bind TGF-β1 and TGF-β3 as well as BMP-9 and can form complexes with the respective receptors. This has been shown to induce Smad1/5/8 activation in endothelial cells [76]. In several types of tumors, overexpression of endoglin is associated with poor prognosis. In a study of mice injected with Lewis lung carcinoma (LLC), continuous overexpression of endoglin was shown to impair the integrity and maturity of tumor vascularization, leading to leaky vessel walls that facilitate intravasation of tumor cells into the bloodstream. Affected animals showed significantly higher numbers of lung metastases compared to wildtype counterparts [77]. Furthermore, in studies of breast cancer, distant metastases were drastically reduced upon simultaneous blockage of angiogenesis through the VEGF inhibitor SU5416 and the endoglin inhibitor TRC105. This was further assessed by means of an endoglin ligand trap in an in vivo model, where metastases were shown to be similarly affected [78]. Similar results were observed when anti-endoglin monoclonal antibodies were used to treat metastasis models of mammary carcinoma. In that study, all tested monoclonal antibodies showed anti-metastatic activities. The authors attributed this to the blockage of angiogenesis of the primary tumor [79]. However, genetic deletion of one allele of endoglin increased metastatic spreading. In a study using RIP1-Tag2 mice as a model, endoglin deficiency led to an increased number of liver metastases [80]. In the same mouse model, a selective VEGFR tyrosine kinase inhibitor, usually protective only in short-term treatment, was able to reduce metastatic burden during long-term treatment. Downregulation of endoglin led to the delayed onset of the resistance to anti-angiogenesis therapy, that normally led to treatment failure in the long term. Therefore, combination of anti-VEGFR treatment and downregulation of endoglin were able to reverse the metastatic effect and suggested a link between VEGF and endoglin signaling. This observation was also translated into therapy in clinical trial phases 1 and 2 in glioblastoma multiforme patients, using a monoclonal antibody against endoglin (TRC105) combined with bevacizumab, an inhibitor of VEGF. Although the combination was well tolerated in patients, the median progression free survival was not prolonged by TRC105 compared to bevacizumab alone [81].

##### β-glycan

The TGF-β receptor type III, also known as β-glycan, is a membrane proteoglycan that binds with high affinity to a variety of molecules such as the three TGF-β isoforms, BMPs, inhibin and FGF. Although β-glycan lacks a functional kinase domain, it influences the TGF-β signaling pathway. For example, β-glycan enhances SMAD2/3 signaling by associating with TGFβRII and thereby presenting the TGF-β isoforms to TGFβRI. Interestingly, the TGF-β isoforms bind with different affinities to TGFβRII, while all bind to TGFβRIII with similar affinity [82]. Due to its multi-faceted role as a co-receptor, TGFβRIII can also suppress SMAD2/3 signaling, for instance by binding to inhibin. Binding inhibin antagonizes the initiation of the SMAD2/3 signaling by preventing the activin-mediated recruitment of TGFβRI and by antagonizing BMP-induced SMAD2/3 signaling [83, 84].

Additionally, the interaction of β-glycan and β-arrestin2 suppresses NFκB signaling by inducing the activation of small GTPase Cdc42, ultimately inhibiting the migration of healthy and breast cancer cells [85, 86].

Besides its co-receptor function, β-glycan can influence signaling pathways depending on its post-translational modifications, such as the presence of heparan and glycosaminoglycans (GAG). Canonical Wnt signaling has been shown to be downregulated by Wnt3a sequestration by these heparan sulfate chains, whereas chondroitin sulfate GAG chains promote Wnt signaling [87].

With its pleiotropic functions, β-glycan can simultaneously influence several pathways, such as the TGF-β signaling pathway, the canonical Wnt pathway and NFκB signaling.

In the clinical context, β-glycan was tested as an adjuvant in 30 breast carcinoma patients, in which it was shown that the administration of soluble β-glycan improved the quality of life [88].

## Concluding remarks

Cancer treatment has made a lot of progress and cancer mortality has declined in the last 30 years [89], a trend that is mainly due to a decreased mortality in four major cancer types: breast, colon, lung and prostate cancers. Although targeted therapy has undoubtedly had a positive impact on cancer treatment, resistance and relapse are observed in advanced cancers [90, 91]. Multiple RTKs inhibition combining inhibitors against various RTKs is hampered by the decrease of concentration of individual compounds to avoid too high side effects [18]. In this scenario, our analysis proposes CD44 and other co-receptors as attractive targets for cancer therapy. Due to its pleiotropic action on cancer cells [23, 32], on the stroma (our own unpublished results) and on signaling from different classes of receptors, CD44 may represent an attractive therapeutic option. The existence of several CD44 isoforms, and their restricted pattern of expression make them candidates with a broad spectrum of action and potentially low side effects. Interestingly, the removal of *Cd44* in the physiological context resulted in mild phenotypes [34, 92] presumably due to the existence of compensatory mechanisms in vivo. In stark contrast, inhibition of CD44 in pathological contexts [32] has a major impact. Therefore, there is a reduced risk of toxicity for physiological processes when targeted with therapeutic intention.

Several efforts have already been made to target CD44v6, one of the CD44 isoforms, in several cancer types in a variety of clinical trials. For instance, an antibody recognizing CD44v6, namely bivatuzumab, was administered together with the anti-tubulin agent mertansine in patients with head and neck squamous cell carcinoma (HNSCC), metastatic breast cancer or esophageal carcinoma [93]. Despite the initially observed benefit, one patient developed lethal skin necrolysis, resulting in a discontinuation of the clinical trial. The toxicity might have been due to mertansine and not to the targeting of CD44v6 [94].

In another clinical trial, solid cancer patients were treated with an antibody against CD44, called RG7356 [95]. Although the humanized monoclonal antibody showed drastic effects in vitro and in vivo in leukemia mouse models, the clinical efficacy of RG7356 was moderate [95].

Another attempt to block CD44v6 was made by our group, as we developed a CD44v6 inhibitory peptide. As previously described, in experimental settings, the administration of the CD44v6 inhibitory peptide drastically decreased primary tumor volume and metastases burden in several mouse models of pancreatic cancer [25]. In a phase Ib trial, patients with advanced or metastatic malignant solid tumors of epithelial origin were treated with a modified version of our CD44v6 peptide as monotherapy (clinicaltrials.gov ID: NCT03009214). However, the outcome of the clinical trial has not yet been published.

The contribution of immune cells to the progression of cancer is widely recognized and several efforts have been made to combine drugs directly targeting cancer cells with immunotherapeutic reagents [96]. For instance, genetically modified patient-derived T cells are redirected against malignant cells by expressing chimeric antigen receptors (CAR). The group of Hanenberg developed a CD44v6 specific CAR T cell that was shown to target specifically tumor-associated CD44v6 of HNSCC cells in vitro. CD44v6 specific CAR T cells underwent multiple clinical trials. For instance, in a first-in-man phase I-IIa starting in 2019 the antitumoral effect of CD44v6 CAR T cells were tested in patients with AML and multiple myeloma (clinicaltrials.gov ID: NCT04097301). Additionally, CAR T cells specific for CD44v6 were also tested in solid cancers including stomach, breast and prostate (clinicaltrials.gov ID: NCT04427449). The combination of multiple CAR T cells that target Her2, GD2 and CD44v6 was tested in the context of breast cancer starting in 2020 (clinicaltrials.gov ID: NCT04430595).

From these various attempts, it is not yet possible to draw a conclusion on the potential efficacy of blocking CD44/CD44v6 alone. However, other aspects of CD44 functions might be useful to test and combination therapies might bring an answer.

Our group is currently addressing the role of CD44v6 in the immunosuppressive microenvironments of pancreatic tumors and their distant metastases in the liver. We are therefore investigating the role of CD44v6 in bone marrow-derived cells (BMDCs) in the context of immunosuppression in the metastatic niche. BMDCs have been shown by Kaplan et al. to form supportive immunosuppressive microenvironments in the distant organs prior the arrival of incoming cancer cells [97, 98]. Our own unpublished data showed that upon the knockout of *Cd44v6* in BMDCs, the immunosuppressive phenotype of BMDCs is turned into a pro-inflammatory nature, as for example, by increased expression levels of *Il1β*, *Tnfα* and *Ifnγ* in vivo. Interestingly, we have also found an increase in *Pdl1* expression. A combinatorial approach in which the PD-L1/PD-1 interaction would be blocked and additionally CD44v6 in vivo is currently under scrutiny.

## Data Availability

No datasets were generated or analysed during the current study.
